# The Neutral—Niche Debate: A Philosophical Perspective

**DOI:** 10.1007/s10441-012-9144-6

**Published:** 2012-02-03

**Authors:** Paul L. Wennekes, James Rosindell, Rampal S. Etienne

**Affiliations:** 1 Community and Conservation Ecology Group, University of Groningen, Groningen, The Netherlands; 2 Institute of Integrative and Comparative Biology, University of Leeds, Leeds, UK

**Keywords:** Dispersal-assembly, Niche-assembly

## Abstract

Ecological communities around the world are under threat while a consensus theory of community structure remains elusive. In the last decade ecologists have struggled with two seemingly opposing theories: niche-based theory that explains diversity with species’ differences and the neutral theory of biodiversity that claims that much of the diversity we observe can be explained without explicitly invoking species’ differences. Although ecologists are increasingly attempting to reconcile these two theories, there is still much resistance against the neutral theory of biodiversity. Here we argue that the dispute between the two theories is a classic example of the dichotomy between philosophical perspectives, realism and instrumentalism. Realism is associated with specific, small-scale and detailed explanations, whereas instrumentalism is linked to general, large-scale, but less precise accounts. Recognizing this will help ecologists get both niche-based and neutral theories in perspective as useful tools for understanding biodiversity patterns.

“In that Empire, the craft of Cartography attained such Perfection that the Map of a Single province covered the space of an entire City, and the Map of the Empire itself an entire Province. In the course of Time, these Extensive maps were found somehow wanting, and so the College of Cartographers evolved a Map of the Empire that was of the same Scale as the Empire and that coincided with it point for point. Less attentive to the Study of Cartography, succeeding Generations came to judge a map of such Magnitude cumbersome, and, not without Irreverence, they abandoned it to the Rigours of sun and Rain. In the western Deserts, tattered Fragments of the Map are still to be found, Sheltering an occasional Beast or beggar; in the whole Nation, no other relic is left of the Discipline of Geography.” (Borges 1975).

## Introduction

In the history of the earth, humans have never before set such high demands on the production capacity of our planet. For example, the effect on biodiversity of our agricultural practices alone is huge; humans now consume about 40% of the total terrestrial production (Tilman 1999) and diverse ecosystems have been replaced by monocultures of agricultural crops such as barley, wheat, corn and rice. Human interference has put biodiversity under threat of habitat degradation and fragmentation, overexploitation, invasive species and climate change (Groom et al. 2005; Mace et al. 2005).

In this context, it is astonishing how little we know about species diversity on earth. For instance, we have named about 1.75 million living species, but current estimates suggest that this is at best only 20% of the true total, and could be as little 3.5% (May 1988; Wilson 1992; Gaston and Spicer 2004). Many species will probably be brought to extinction before they have even been named, let alone their function in the ecosystem understood. Solid theories explaining the mechanisms underlying biodiversity are therefore urgently needed.

The classical explanation of biodiversity is that through evolution every species has acquired a unique set of traits that allow it to be adapted to a particular environment (abiotic as well as biotic): it occupies a unique niche. While the concept of niche is difficult to define (Chase and Leibold 2003; Soberón and Nakamura 2009), the major idea behind this classical theory is clear: species are fundamentally different and these differences allow them to coexist. The neutral theory of biodiversity (from hereon referred to as just ‘neutral theory’) does not emphasize species differences; it leaves them out by assuming the functional equivalence of all (trophically similar) individuals in the ecological community under consideration. Species can be different as long as those differences do not entail functional differences. Neutral theory explains diversity as a stochastic balance between speciation and extinction on continental scales, or immigration and extinction on local scales (Hubbell 2001; Leibold and McPeek 2006).

Neutral ideas are almost as old as the niche concept (for a brief historical overview, see Etienne and Alonso 2007), but neutral theory never received much attention until it was promoted and developed extensively in “The unified neutral theory of biodiversity and biogeography” (Hubbell 2001). Since then neutral theory had a mixed reception amongst biologists (for reviews see Alonso et al. 2006; Leigh 2007; Leigh et al. 2010; Rosindell et al. 2011). The utility of neutral theory is now becoming increasingly recognized and many are striving to reconcile the neutral and niche-based theories in a single unified framework (e.g. Gravel et al. 2006, 2011; Leibold and McPeek 2006; Adler et al. 2007; Herault 2007; Vellend 2010; Chisholm and Pacala 2010; Haegeman and Etienne 2011). For example, it is realized that both stabilizing (niche-based) forces, where a species limits itself more than it does others, and equalizing (neutral) forces that reduce fitness differences between species play a role in maintaining diversity (Chesson 2000; Adler et al. 2007). Nevertheless, the resistance against the neutral theory of biodiversity remains strong and attempts are frequently made to falsify or reject neutral theory, albeit on very different grounds (McGill 2003; Wootton 2005; Dornelas et al. 2006; McGill et al. 2006; Clark 2008, 2010).

In this article we discuss three general categories of criticism against neutral theory.

1. Neutral theory starts from false assumptions.
2. Neutral theory makes false claims.
3. Neutral theory is not a good theory because it does not fit with the philosophical paradigm of realism.

According to realism, all entities and assumptions in a theory should be real and true for the theory to work (Putnam 1975). In contrast, according to instrumentalism, a theory’s utility is more important than its literal truth. We argue that the dispute between supporters of niche-based theory and neutral theory is a classic example of a clash between two philosophical perspectives: realism and instrumentalism, respectively. The arguments are tightly linked with the philosophical preference for either general but vague versus specific and detailed models (Levins 1966). An ideal model explains or predicts much but requires few assumptions and parameters, but such ideal models do not often exist. Adding more details usually requires sacrificing generality and generality only comes at the cost of simplifying assumptions. The ‘general, large-scale, but vague’ models need few assumptions and parameters, yet may still provide a good approximation, while the ‘specific, small-scale and precise’ models require more parameters or have more assumptions. Science is an abstraction and it is subjective; scientists leave out elements that they consider to be unimportant, and keep the remainder, but different scientists will have different opinions on what the important elements are.

In resolving the dispute it is necessary that implicit assumptions in the instrumentalism-realism debate are made explicit so that it is clear whether proponents and opponents are discussing the same matters, or arguing from different premises. We believe that simply recognizing that the debate is an instance of a classic reoccurring philosophical debate will aid in cutting past the niche versus neutral arguments and accepting different perspectives as useful tools in understanding biodiversity patterns. We first describe the instrumentalism versus realism debate in isolation and then discuss the criticisms of neutral theory in this context. We defend neutral theory from an instrumentalist perspective. This differs from the way in which the theory is typically and historically defended; we argue for the utility of neutral theory, free from any historical burden.

## Instrumentalism Versus Realism

The struggle between instrumentalist and realist approaches is a common theme in any field of science (Esfeld 2001), but there has been an emphasis on realist approaches for the good latter half of the twentieth century, especially in ecology. Realist accounts of natural phenomena point to content and assumptions of a model as being more important than the predictive value or usefulness of the model. In the realist’s view, a true model will always perform well, precisely because it is true. In contrast, instrumentalists emphasize the predictive value and other uses of a model; the literal truth of the model is not an issue.

In ecology there is also a preference for specific, typically small-scale and detailed models, perhaps because these are more practical to construct in a way that is satisfactory from a realist perspective. General, large-scale but less precise models are usually waved away as too simplistic even if their predictions are correct. These models will often be framed in terms of stochasticity (Nelson 1985) whereas a specific (small-scale) and detailed model will often give a more deterministic account of the studied phenomena.

Stochasticity in a model may be a real property of the world or just a consequence of the presence of more unknown variables. In the latter case stochasticity is a property of the model rather than a reflection of reality and the dichotomy moves from an ‘*ontological*’ dichotomy where there are two contrasting views of the *real workings of nature*, to an ‘*epistemological*’ dichotomy where there are two ways to best *describe, understand, model or explain* the workings of nature. This is the abovementioned dichotomy between realism and instrumentalism once again.

We want to stress that realism and instrumentalism are relative terms and so are ‘small scales’ and ‘large scales’: if the species composition in a tropical rainforest is the subject of study, models based on the differences between species and individuals might be considered small-scale but if the behavior of an individual organism is the subject, speaking in terms of individuals might be considered a large-scale approach. A small-scale approach might in this case involve a closer analysis of the individual’s cells or organs.

There are many disputes across the fields of science that in the end reduce to this simple distinction, particularly in the areas of personality, brains and behavior. Properties of persons, such as thinking and experiencing are often held to be incompatible with lower-level properties of neurons, like electrical discharge, that are said to determine these higher-order properties. In the philosophy of mind, this has even become known as the ‘hard problem’. The hard problem consists of a seeming contradiction to a physicalist account of the brain and the fact that we have phenomenal consciousness. Different opinions are available on the status of this ‘explanatory gap’. Some philosophers take the explanatory gap as proof that physicalism is false (Chalmers 1995). Others try to denounce our experiences to try and maintain a monistic worldview (Churchland 1981). Neither approach seems very satisfactory; the former leads towards a dualistic worldview, whereas the latter leads towards denouncing patterns and processes that clearly exist.

Following a strictly realist line of thought, the problem arises that if both explanations are true, we have several explanations for essentially the same phenomena and one of them will have to go. However, this is a black-or-white view of a scientific problem. It is argued by philosophers such as Wittgenstein (1954), Hacker (2003) and Putnam (2000), that trying to solve a seeming discrepancy between several levels of explanation is in fact a misuse of language: exactly because thinking is a property of persons and electrical discharge is a property of neurons, there is no contradiction between saying that a person is thinking or that a neuron is releasing an electrical charge. It depends on the (scale of the) question that we wish to answer which approach is more appropriate. This means that certain uses of language must be ruled out; because thinking is a human property and occurs on a certain explanatory level where people and personalities play a role, it is nonsensical to say something like ‘my neurons are thinking ‘or ‘I’m discharging my neurons’. It would be strange to intertwine the different levels of explanation in such a way; it would be like saying ‘my arm is playing tennis’.

## Niche-Based and Neutral Models

The traditional approach to understanding community structure is the niche-assembly perspective, Hubbell contrasts it with the dispersal-assembly perspective to which his neutral theory belongs as follows:

The niche-assembly perspective asserts that ecological communities are limited membership assemblages of species that coexist at equilibrium under strict niche partitioning of resources. […] The dispersal-assembly perspective asserts that ecological communities are open, continuously changing, non-equilibrium assemblages of species whose presence, absence, and relative abundance are governed by random speciation and dispersal, ecological drift, and extinction. (Hubbell 2001 p. 29)

The niche-assembly perspective is the small-scale, but precise approach; the composition of an ecosystem in this view is just a higher-level summation of processes taking place at the individual level, which can be studied separately.

The niche-assembly perspective asserts that an ecological community is made up of a limited number of niches, each occupied by a single species. Although there has been no consensus on a full definition for the word ‘niche’, a general description of the concept illustrates the key underlying idea: a niche is a (hyper-)volume in a set of dimensions which expresses the capability of a species to exploit resources. (Hutchinson 1959) These dimensions can be traditional dimensions such as time and space, but they can also represent factors such as prey size, temperature, moisture levels and nutrient availability. The niche of an organism can be seen as the n-dimensional space in which a species can live, n being the number of factors, which are considered relevant for the survival of the species. Each species has a fundamental niche, which is the n-dimensional space in which they can theoretically survive. However, most species will have competitors whose niches may partially or wholly overlap their own. The species that is more efficient in the overlapping part of their fundamental niches will, in a process which is known as niche partitioning or competitive exclusion, attempt to exclude the other species by outcompeting them. This can lead to two scenarios; either one species will win and the other will go extinct, or, in a reaction to the evolutionary pressure, one or both species may undergo a change in specialization away from the contested part (character displacement), effectively reducing the niche-overlap between the two species, thus avoiding extinction. The realized niche of a species is the part of their fundamental niche that they actually occupy. For simplicity, we have explained niches in terms of competition, but evidently there are other interactions (e.g. trophic interactions) that also reduce a species’ fundamental niche.

The niche-assembly perspective is widely used throughout ecology, but it has its drawbacks. In many cases, it seems to struggle with satisfactorily explaining coexistence of many species, a problem known as the Paradox of the plankton (Hutchinson 1961). Although various clever niche-based solutions to this paradox have been offered, these solutions are often complex, and one may wonder whether all this complexity is really needed. These solutions often propose the existence of previously unobserved niche axes to explain species coexistence, potentially making the niche assembly perspective unfalsifiable, because there can always be an argument for another undiscovered niche axis.

The dispersal-assembly perspective focuses more on large-scale processes, both temporal and spatial. Instead of explaining the composition of an ecosystem in terms of competitive differences and niche-partitioning, it considers the structure of an ecological system as a result of dispersal. No longer does the explanation of why a certain tree is standing at a particular location have to be answered with “this particular spot suits its niche best”. The answer from a dispersal-limited perspective is “this individual was able to disperse to that spot, or accidentally landed here”. Principles such as ecological drift and local extinction are considered to be the key driving factors of ecological systems in the dispersal limited approach.

Neutral theory, as an exponent of the dispersal-assembly perspective, is not meant to be an attack of niche-assembly theory. Rather, it highlights the importance of other processes such as dispersal limitation and ecological drift that are often neglected (Etienne and Alonso 2005; Etienne 2007). This is fully in line with the instrumentalist philosophy, because the instrumentalist allows for and sees merit in all approaches that work and/or have utility, including realist approaches, and the instrumentalist does not necessarily limit himself to only one approach. Neutral theory builds a community from many individuals interacting in simple ways, but in neutral theory a large part of the explanatory value comes from emergent behavior that would not at first glance be expected to emerge from these simple interactions. For the instrumentalist, these emergent properties can be studied on their own, without having to deal with difficult questions about whether these are real, or which level of explanation has ontological primacy; both the lower and the higher levels of explanation are tools, of which the validity is only dependent on their usefulness. Evidently, Neutralists such as Hubbell recognize the existence of niches and their importance in ecosystems. What the question should be is: what is the relative importance of niche-assembly and dispersal-assembly in determining the composition of different ecosystems? Both will play some role, but their relative importance is likely to be very different depending on the environment chosen and the taxonomic group under consideration (Adler et al. 2007).

## Criticisms of Neutral Theory

### Neutral Theory Starts from False Assumptions

McGill et al. (2006) aptly summarize the aversion to neutral theory as follows: “neutral theories of biodiversity assert that all individuals of all species are competitively identical. […] This contradicts 100 years of community ecology”. Evidently, neutral theory does start from assumptions that are partly false, but so do many other theories for reasons of simplicity, elegance and practicality (e.g. metapopulation theory of Levins 1969; see Etienne 2000, 2002; or the metabolic theory of ecology, of Brown et al. 2004; see Etienne et al. 2006; Apol et al. 2008). The most obvious example in the case of neutral theory is the assumption of neutrality: equivalence between individuals belonging to different species. However, other auxiliary assumptions exist and can be relaxed (Leigh 2007), for example concerning speciation (Etienne et al. 2007a; Etienne and Haegeman 2011; Rosindell et al. 2010), spatial structure (Rosindell and Cornell 2007, 2009) and the zero-sum rule (Etienne et al. 2007b; Haegeman and Etienne 2008), and this often does not affect the theory’s predictions or produces predictions that match observations better.

Although neutral theory does assert, for the sake of simplicity, that all individuals are equivalent, this should not be regarded as a weak spot of the theory. Rather, it is its strongest asset: it makes tractable models that describe the dynamics of an ecological system without adaptation. The only reason that neutrality is regarded as an extra assumption is that so many other models include non-neutral details. To say that ‘all individuals of different species are ecologically equivalent’ is arguably not an assumption, but simply a lack of detail about the specifics of ecological interactions given by competing theories. If a lack of information is generally considered as an assumption, the list of ‘assumptions’ for almost any model can readily become long and preposterous. For example imagine the phrase ‘this model assumes that no evolution occurs and no new species evolve, that organisms move unrestricted about the ecosystem, that conspecific individuals have no genetic differences, that seasonality and the weather have no influence on the ecosystem, that species never go extinct, that parasites do not exist,…’. We do not generally provide such a list with models. Instead we are more likely to see ‘seasonality is modeled by assuming…’ or simply no mention of seasonality. With the construction of such a list of assumptions any person could, with careful rhetoric, make any model appear worthless. Compare the neutral theory of community ecology with that of population genetics: examples of evolution by natural selection are not generally regarded as proof that genetic drift does not exist. Rather, models based only on genetic drift describe the dynamics of a population’s genetic diversity in absence of natural selection, and a baseline against which to compare data of cases where natural selection is present.

### Neutral Theory Makes False Claims

From an instrumentalist perspective, false assumptions are not a proper reason to dismiss a theory; theories should not be evaluated based on their assumptions but on their predictions. Friedman (1966) put it even more strongly:

“Truly important and significant hypotheses will be found to have ‘assumptions‘that are wildly inaccurate descriptive representations of reality, and, in general, the more significant the theory, the more unrealistic the assumptions (in this sense). […] The reason is simple. A hypothesis is important if it ‘explains‘much by little, that is, if it abstracts the common and crucial elements from the mass of complex and detailed circumstances surrounding the phenomena to be explained and permits valid predictions on the basis of them alone.” (Friedman 1966)

Ricklefs (2003) was one of the first to cut past the assumptions, looking instead at neutral theory’s predictions, which he deemed to be not very impressive. Some of this original criticism (and the similar criticism of Nee 2005) can, however, be attributed to immature elements of the theory at the time. Rosindell et al. (2010) solved some of the problems that Ricklefs raised by making speciation in the neutral model more realistic. McGill (2003) and McGill et al. (2006), even though they consider neutral theory’s assumptions to be false, also (in line with Friedman’s philosophy) evaluated its predictions and thus made use of the theory in the proper scientific way. From an extensive literature review of tests of neutral theory vs. niche-assembly theory they concluded that there is little evidence to support neutral theory. Similarly, Dornelas et al. (2006) claimed that the data they find in coral reefs does not match the predictions made by neutral theory. Whilst some of these failures of neutral theory may again be attributed to auxiliary assumptions (in the case of the corals, Volkov et al. 2007 show that a modified neutral model can in fact produce results that fit the observed coral data well, although they require low immigration rates to achieve this, which are considered unrealistic for corals), there are of course cases where neutral theory’s predictions will not match observations. And even if they do, this does not necessarily imply that niche-based mechanisms are unimportant, because neutral pattern does not imply neutral process (see for example Du et al. 2011).

From a classical instrumentalist perspective, failure of the neutral theory (even after it has matured) should lead to rejection of the theory. We agree with this procedure, but we argue that there is a second instrumentalist role to play for neutral theory. Failure to match empirical patterns does not mean that theory is useless. Instead it means that the theory has done its job in highlighting that something other than dispersal limitation or ecological drift plays an important role *that is visible* *in the data being studied* (as opposed to a role that is *not visible in the data being studied, which is probably played by many (non*-*neutral) factors)* (Pearson and Gardner 1997; Rosindell et al. 2012). Although this may sound like we are attempting to make neutral theory invincible in some way, it is crucially the usefulness of neutral theory as a tool and not the claim that the world is neutral that we are defending here. When neutral theory fails, it does so in an informative manner: we must extend it to properly explain the system. If neutral theory succeeds, we may not need to look further for understanding the system, or we should question whether our data set is informative enough. For example, in the case of coral diversity we argue that refuting the theory should not be interpreted too negatively. Failure of neutral theory shows that there must be alternative mechanisms. Dornelas et al. (2006) hypothesize that environmental heterogeneity may be playing an important role.

Thus, neutral theory has a dual instrumentalist function: like any other theory, it can be used to predict patterns, but unlike many other theories, it is well positioned to act as a starting point, a baseline model to which one can later add more ecological mechanisms. It is exactly when it makes predictions that are not supported by empirical data that this second role is played. The following analogy illustrates our point. If one uses a gauge to test tire pressures, and finds the tire is under filled, the pressure gauge has not failed, but it has succeeded and done its job, even though the result of the test it was used for was a failure.

Attacks on neutral theory because of its inability to match some empirical data fail to see the role of the theory in a broader context; that of one theory amongst others which can be of practical importance to highlight different aspects of a particular problem that usually go unnoticed or receive less attention than they should. Niche differences have long intrigued biologists, and rightfully so, but they are not the only factors governing diversity patterns in ecological communities (Vellend 2010).

In summary, we argue that neutral theory should not be seen as just a null model in the statistical sense, one that can be rejected, but rather as a baseline model that contains necessary ingredients that more advanced models should often also contain (similar to the role of the neutral theory of population genetics that emphasized the omnipresence of genetic drift). We believe that starting from neutral theory is much easier than starting from a model that assumes niche differentiation from the outset. There might be other simple starting points as well. In this context, McGill (2010) identified three assumptions (which he calls “rules”) from which most of the (approximately) correct predictions of neutral theory and five other ‘unified’ theories can be derived: (1) intraspecifically individuals are clumped together; (2) interspecifically global or regional abundance varies according to a hollow curve distribution; and (3) interspecifically individuals are placed without regard to individuals of other species. These rules are statistical rather than mechanistic, but for the instrumentalist, this does not matter.

### Neutral Theory is Not a Good Theory Because it Does Not Fit with the Philosophical Paradigm of Realism

We argue that this opinion about neutral theory is often held by critics, at least implicitly or without realizing it. This is not an argument that is often expressed explicitly, and consequently it is hard to settle. A good example of a realist plea is the opinion paper by Clark (2008), in which he argues that neutral theory does not contain a real process because its stochastic elements that are perceived as neutral forces are simply standing in for smaller scale non-neutral processes that are not modeled explicitly. It appears that Clark (2008) believes neutral theory to be a contrasting view on the real workings of the world, and hence he charts the ontological dichotomy. He goes on to argue that stochastic elements only exist in models and although he admits utility of such models for understanding and explanation, he argues that there are more promising alternative directions. This illustrates the epistemological dichotomy.

The dispute between niche-based and neutral theory is quite similar to the situation between the neurosciences and folk-psychological accounts of behavior. Niche-assembly is in itself a more realist theory than neutral theory; it focuses on detailed interactions between individuals and species and from this ‘real’ base builds up a picture of the ecological community as a whole. Neutral theory partly works top-down: local community structure is determined, to a considerable extent, by processes at the metacommunity level, for the simple reason that describing matters in that way makes them easier to understand. There is no ontological primacy given to either level.

On a more abstract level there is the question of whether we should try to choose between two seemingly conflicting theories, or try to reconcile them. This is in itself a choice between a realist and an instrumentalist approach, which explains why it is no wonder that the former option—conflict—is often preferred by those supporting niche-assembly theory (although significant reconciliatory attempts are also made by some proponents of niche-assembly; e.g. Leibold and McPeek 2006) and the latter option—reconciliation—is preferred by those tolerating neutral theory. Someone who takes a realist or instrumentalist approach in one area will likely have the same preference in another area.

The main reason why arguments about testing neutral against niche theory sound so convincing is that the realist story behind these tests is so appealing. We claim that denying the possibility of reconciliation between the two theories is ultimately counterproductive to getting a full understanding of biodiversity patterns and how ecosystems work. Even though a realist approach can advance science, so can an instrumentalist approach which should merit its application to understanding the origin and maintenance of biodiversity.

There has always been a strong realist tendency in the natural sciences and in biology in particular. There is value in this; a realist approach means that there cannot be two competing explanations for a single phenomenon and science should progress by decisive tests eliminating false alternatives (Platt 1964). Ultimately, the realist approach leads to reducing the problem into simpler sub-problems that can then be solved one by one, eventually being recombined to solve the original problem in its entirety. This approach does not work for every problem however, and over-applying it could make us miss emergent patterns that are only clearly observable when looking at the system as a whole (Quinn and Dunham 1983).

## Conclusion

**Fig. 1:**
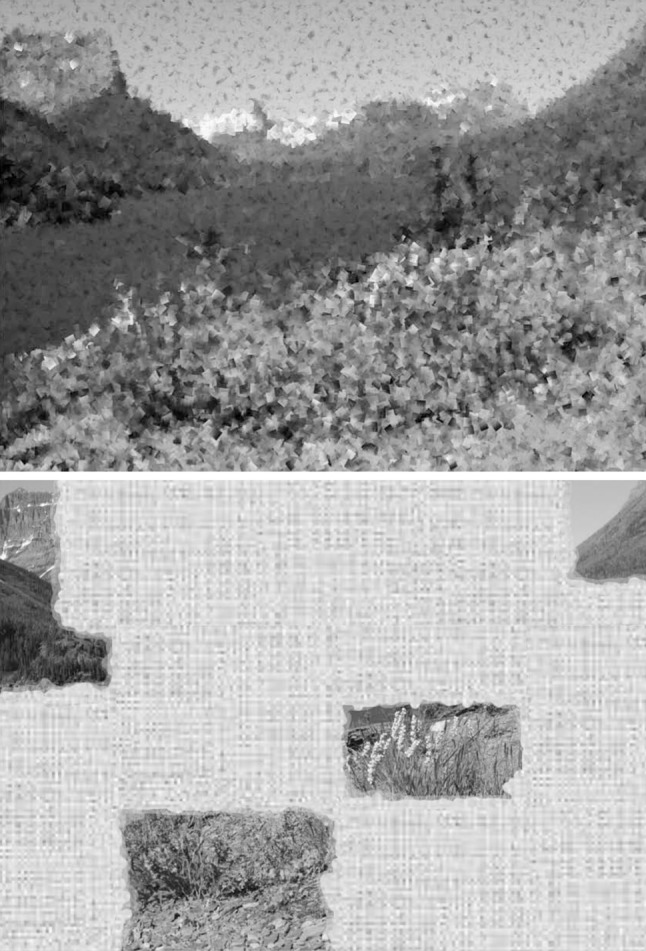
A natural landscape of wildflowers, evergreen trees and mountains photographed at Glacier National Park in Montana USA. The top image has had its details removed to illustrate a half completed instrumentalist painting of the landscape. The bottom image contains all details but only part of the picture is visible, the rest is blank canvas: this is the half-completed realist painting. In this example we are reminded of the difference between ‘realism’ and ‘impressionism’ in art where the latter was a more recent advance that was initially met with substantial criticism

Science is biased towards realism and the ‘specific small-scale but detailed’ approach, because the realist approach has been successful. The worldview where quarks make up atoms and atoms make up molecules works extremely well and ultimately, every phenomenon seems to be explained by the interactions between real entities. So where does the instrumentalist approach fit in? The instrumentalist argues first that a theory need not make correct assumptions to be useful as a predictive theory, second, that it need not make correct predictions to be useful as a guide towards further theory development and third, that it is impractical to specify details from the outset. A perfect painter could make a beautiful painting by completely finishing the top left square inch, then completely finishing the next square inch and so on (for the more digitally-minded: this is similar to how a printer produces images). In reality however, it makes more sense to block in with pencil what overall layout is wanted, then paint the big parts, then add details (in the way most human artists work). The end result is in theory the same in either case: we have a beautiful painting. However, a half-finished painting would look very different depending on the method the painter used: the instrumentalist painter would give an overall impression, which would however lack detail, while the realist painter would have a large area of blank canvas but the finished bits would contain perfect detail (see Fig. 1). Neutral theory paints a picture of biodiversity in the former sense, while niche theory, with detailed models for specific systems, pictures it in the latter sense. Niche theory provides stunningly beautiful details, but much of the canvas is still blank and it is not even clear how large the canvas really is. A combined approach would be the most useful way to go about painting the image. Debatably, it may also be the only way to end up with the perfect picture.

The painting metaphor clearly illustrates the instrumentalism-realism dichotomy, as well as the dichotomy between the approaches from different scales. For a realist, who focuses on the small scale, neutral theory is appalling as it leaves out real species differences, that is, it essentially ignores the trees in a tropical forest. For an instrumentalist, focusing on the big picture, neutral theory is a potentially useful tool to avoid the danger that we cannot see the forest for the trees.

A scientific account of any system should be clear and consistent in its language use. Proponents of different approaches should recognize that there are different explanatory levels—different ways of painting images, if you will—and should be clear in their definitions, because this prevents faulty use of language and thereby false dichotomies: it would be easier to see whether two different theories about the same system are actually mutually exclusive or if they can be used in conjunction. If this were to be implemented in the field of biogeography and biodiversity, more research effort could be used to understand the patterns and processes we are so interested in, instead of battling each other over dichotomies that are not, in fact, dichotomies.

Science is much like map-making: providing a comprehensive summary of a system under study. Long before Borges (1975), Lewis Carroll (1893) realized that this is not trivial:

“That’s another thing we’ve learned from your Nation,” said Mein Herr, “map-making. But we’ve carried it much further than you. What do you consider the largest map that would be really useful?”

“About six inches to the mile.”

“Only six inches!” exclaimed Mein Herr. “We very soon got to six yards to the mile. Then we tried a hundred yards to the mile. And then came the grandest idea of all! We actually made a map of the country, on the scale of a mile to the mile!”

“Have you used it much?” I enquired.

“It has never been spread out, yet,” said Mein Herr, “the farmers objected: they said it would cover the whole country, and shut out the sunlight! So we now use the country itself, as its own map, and I assure you it does nearly as well.” (Carroll, 1893)

## References

[CR1] Adler PB, HilleRisLambers J, Levine JM (2007). A niche for neutrality. Ecol Lett.

[CR78] Alonso D, Etienne RS, McKane AJ (2006) The merits of neutral theory. Trend Ecol Evol 21:451–457 10.1016/j.tree.2006.03.019 16766082

[CR2] Apol MEF, Etienne RS, Olff H (2008). Revisiting the evolutionary origin of allometric metabolic scaling in biology. Funct Ecol.

[CR5] Borges JL (1975) On exactitude in science. In: A universal history of infamy. Penguin Books, London. Translated by Norman Thomas di Giovanni

[CR7] Brown JH, Gillooly JF, Allen AP, Savage VM, West GB (2004). Toward a metabolic theory of ecology. Ecology.

[CR10] Carroll L (1893). Sylvie and Bruno concluded.

[CR11] Chalmers DJ (1995). Facing up to the problem of consciousness. J Conscious Stud.

[CR12] Chase JM, Leibold M (2003). Ecological niches: linking classical and contemporary approaches.

[CR13] Chesson P (2000). Mechanisms of maintenance of species diversity. Annu Rev Ecol Syst.

[CR14] Chisholm RA, Pacala SW (2010) Niche and neutral models predict asymptotically equivalent species abundance distributions in high-diversity ecological communities. In: Proceedings of the national academy of sciences of the USA (in press) 10.1073/pnas.1009387107 PMC2936647 20733073

[CR15] Churchland PM (1981). Eliminative materialism and the propositional attitudes. J Philos.

[CR16] Clark JS (2008). Beyond neutral science. Trends Ecol Evol.

[CR17] Clark JS (2010). Individuals and the variation needed for high species diversity in forest trees. Science.

[CR19] Dornelas M, Connolly SR, Hughes TP (2006). Coral reef diversity refutes the neutral theory of biodiversity. Nature.

[CR20] Du X, Zhou S, Etienne RS (2011). Negative density dependence can offset the effect of species competitive asymmetry: a niche-based mechanism for neutral-like patterns. J Theor Biol.

[CR21] Esfeld M (2001). Holism in philosophy of mind and philosophy of physics.

[CR22] Etienne RS (2000). Local populations of different sizes, mechanistic rescue effect and patch preference in the Levins metapopulation model. Bull Math Biol.

[CR23] Etienne RS (2002). A scrutiny of the Levins metapopulation model. Comments Theor Biol.

[CR24] Etienne RS (2007). A neutral sampling formula for multiple samples and an “exact” test of neutrality. Ecol Lett.

[CR25] Etienne RS, Alonso D (2005). A dispersal-limited sampling theory for species and alleles. Ecol Lett.

[CR26] Etienne RS, Alonso D (2007). Neutral community theory: how stochasticity and dispersal-limitation can explain species coexistence. J Stat Phys.

[CR27] Etienne RS, Haegeman B (2011). The neutral theory of biodiversity with random fission speciation. Theor Ecol.

[CR28] Etienne RS, Apol MEF, Olff H (2006). Demystifying the West, Brown & Enquist model of the allometry of metabolism. Funct Ecol.

[CR29] Etienne RS, Alonso D, McKane AJ (2007). The zero-sum assumption in neutral biodiversity theory. J Theor Biol.

[CR30] Etienne RS, Apol MEF, Olff H, Weissing FJ (2007). Modes of speciation and the neutral theory of biodiversity. Oikos.

[CR31] Friedman M (1966) The methodology of positive economics. Pages 3–16, 30–43 in Essays in positive economics. University of Chicago Press, Chicago

[CR32] Gaston KJ, Spicer I (2004). Biodiversity: an introduction.

[CR33] Gravel D, Canham CD, Beaudet M, Messier C (2006). Reconciling niche and neutrality: the continuum hypothesis. Ecol Lett.

[CR34] Gravel D, Guichard F, Hochberg MH (2011). Coexistence in a variable world. Ecol Lett.

[CR35] Groom JG, Meffe GK, Carroll CR (2005). Principles of conservation biology.

[CR36] Hacker P (2003). Philosophical foundations of neuroscience.

[CR37] Haegeman B, Etienne RS (2008). Relaxing the zero-sum assumption in neutral biodiversity theory. J Theor Biol.

[CR38] Haegeman B, Etienne RS (2011). Independent species in independent niches behave neutrally. Oikos.

[CR39] Herault B (2007). Reconciling niche and neutrality through the emergent group approach. Perspect Plant Ecol Evol Syst.

[CR40] Hubbell SP (2001). The unified neutral theory of biodiversity and biogeography.

[CR41] Hutchinson GE (1959). Hommage to Santa Rosaria, or why are there so many kinds of animals?. Am Nat.

[CR42] Hutchinson GE (1961). The paradox of the plankton. Am Nat.

[CR43] Leibold M, McPeek M (2006). Coexistence of the niche and neutral perspectives in community ecology. Ecology.

[CR44] Leigh EG (2007). The neutral theory: a historical perspective. J Evol Biol.

[CR45] Leigh EG, Rosindell J, Etienne RS (2010). Unified neutral theory of biodiversity and biogeography. Scholarpedia.

[CR46] Levins R (1966). The strategy of model building in population biology. Am Sci.

[CR47] Levins R (1969). Some demographic and genetic consequences of environmental heterogeneity for biological control. Bull Entomol Soc Am.

[CR51] Mace G, Masundire H, Baillie J (2005) Millennium ecosystem assessment: ecosystems and human well-being. http://www.maweb.org/en/index.aspx

[CR52] May RM (1988). How many species are there on earth?. Science.

[CR54] McGill BJ (2003). A test of the unified neutral theory of biodiversity. Nature.

[CR55] McGill BJ (2010). Towards a unification of unified theories of biodiversity. Ecol Lett.

[CR56] McGill BJ, Maurer BA, Weiser MD (2006). Empirical evaluation of neutral theory. Ecology.

[CR57] Nee S (2005). The neutral theory of biodiversity: do the numbers add up?. Funct Ecol.

[CR58] Nelson E (1985). Quantum fluctuations.

[CR76] Pearson SM, Gardner RH (1997) Neutral models: useful tools for understanding landscape patterns. In: Bissonette JA (ed) Wildlife and landscape ecology: effects of pattern and scale, New York, Springer , pp 215–230

[CR59] Platt JR (1964). Strong inference. Science.

[CR60] Putnam H (1975). Mathematics, matter and method.

[CR61] Putnam H (2000). The threefold cord: mind, body, and world.

[CR79] Quinn JF, Dunham AE (1983) On hypothesis testing in ecology and evolution. Am Nat 122:602–617

[CR62] Ricklefs RE (2003). A comment on Hubbell’s zero-sum ecological drift model. Oikos.

[CR63] Rosindell J, Cornell SJ (2007). Species-area relationships from a spatially explicit neutral model in an infinite landscape. Ecol Lett.

[CR64] Rosindell J, Cornell SJ (2009). Species-area curves, neutral models and long distance dispersal. Ecology.

[CR65] Rosindell J, Cornell SJ, Hubbell SP, Etienne RS (2010). Protracted speciation revitalizes the neutral theory of biodiversity. Ecol Lett.

[CR66] Rosindell J, Hubbell SP, Etienne RS (2011). The unified neutral theory of biodiversity and biogeography at age ten. Trends Ecol Evol.

[CR77] Rosindell J, Hubbell SP, He F, Harmon LJ, Etienne RS (2012) The case for ecological neutral theory. Trends Ecol Evol. doi:10.1016/j.tree.2012.01.004 10.1016/j.tree.2012.01.004 22341498

[CR67] Soberón J, Nakamura M (2009). Niches and distributional areas: concepts, methods, and assumptions. Proc Natl Acad Sci USA.

[CR70] Tilman D (1999). Global environmental impacts of agricultural expansions: the need for sustainable and efficient practices. Proc Natl Acad Sci USA.

[CR71] Vellend M (2010). Conceptual synthesis in community ecology. Q Rev Biol.

[CR72] Volkov I, Banavar JR, Hubbell SP, Maritan A (2007). Patterns of relative species abundance in rainforests and coral reefs. Nature.

[CR73] Wilson EO (1992). The diversity of life.

[CR74] Wittgenstein L (1954). Philosophical investigations.

[CR75] Wootton JT (2005). Field parameterization and experimental test of the neutral theory of biodiversity. Nature.

